# Long-Term Persistence of Serum-Specific Anti-Chikungunya IgM Antibody - A Case Series of Brazilian Patients

**DOI:** 10.1590/0037-8682-0855-2020

**Published:** 2021-04-12

**Authors:** Denise Maria do Nascimento Costa, Maria Rosângela Cunha Duarte Coêlho, Pedro Alves da Cruz Gouveia, Luan Araújo Bezerra, Claudia Diniz Lopes Marques, Angela Luzia Branco Pinto Duarte, Lucila Maria Valente, Vera Magalhães

**Affiliations:** 1 Universidade Federal de Pernambuco, Hospital das Clínicas, Recife, PE, Brasil.; 2 Universidade Federal de Pernambuco, Departamento de Virologia, Laboratório de Imunopatologia Keizo Asami, Recife, PE, Brasil.; 3 Universidade Federal de Pernambuco, Centro de Biociências, Departamento de Fisiologia e Farmacologia, Recife, PE, Brasil.

**Keywords:** Chikungunya fever, Immunoglobulin M, Arthritis

## Abstract

The persistence of serum-specific anti-chikungunya IgM antibodies (CHIKV-IgM) can vary after chikungunya fever (CHIK) infection. However, the factors related to its production are not yet known. We described a case series drawn up from data collected from 57 patients between 12 and 36 months after the acute phase of CHIK infection in Northeastern Brazil. CHIKV-IgM was detectable in 7/57 (12.3%) patients after 28.3 months of infection. No frequency differences in chronic musculoskeletal manifestations and underlying conditions were detected between patients with or without CHIKV-IgM. CHIKV-IgM was detected for up to 35 months in Brazilian patients after CHIK infection.

## INTRODUCTION

Chikungunya virus (CHIKV) is an alphavirus of the *Togaviridae* family, transmitted by Aedes mosquitoes and causes chikungunya fever (CHIK)^1^. Although it was first described in 1952, CHIKV was first observed in Brazil in 2014, and during the subsequent epidemic, more than 400.000 cases were reported^1^ ^,^ ^2^. In Northeastern Brazil, the state of Pernambuco recorded a higher number of deaths and an incidence rate almost four times higher than the rest of the country^2^. This disparity has raised questions regarding the course of CHIK in our intensely admixed population^3^.

It is known that in the viremia phase, CHIKV causes B-cell activation and early production of immunoglobulins, with the detection of serum-specific anti-chikungunya IgM antibodies (CHIKV-IgM) expected from the onset of infection until approximately three months after infection^4^. Studies conducted in different regions of the world have demonstrated that the persistence of CHIKV-IgM may be quite variable. However, most studies have been limited to a period of up to 18 months after infection, and none have included Brazilian patients^4^ ^-^ ^10^.

Although this antibody does not present an established pathogenic mechanism, it has been questioned whether there is a relationship between the serum persistence of this immunoglobulin and chronic joint symptoms in CHIK^11^ ^,^ ^12^. Notably, the reason that some patients develop chronic arthralgia is poorly understood. Chronic forms of CHIK occur in approximately 10−60% of patients and may cause persistent, severe disability, which significantly decreases quality of life^1^. Some authors have controversially suggested that the chronicity of CHIK symptoms may be related to the presence of pre-existent comorbidities^13^ ^,^ ^14^. Thus, this case series aimed to identify the frequency of detectable serum CHIKV-IgM in patients with chronic symptoms from 12 to 36 months after CHIKV infection in a Brazilian university hospital, describing the chronic musculoskeletal manifestations and previous comorbidities of those with positive antibodies.

## CASE REPORT

From December 2018 to July 2019, a case series was prepared from data collected from 57 patients diagnosed with CHIK, who were previously evaluated in a cross-sectional study, between 12 and 36 months after the acute phase of infection. These patients were followed-up in a specific outpatient clinic for people with chronic symptoms after CHIK infection at the Hospital das Clínicas at the Universidade Federal de Pernambuco, Northeastern Brazil. A diagnosis of CHIK was established based on clinical and epidemiological criteria and confirmed by the detection of serum CHIKV-IgM or IgG by enzyme-linked immunosorbent assay (ELISA). The study was approved by the local ethics committee, and consent forms were obtained from all patients before inclusion in the study. 

At the time of the evaluation, blood samples were collected and tested for CHIKV-IgM using a commercially available qualitative ELISA, following the manufacturer's instructions (anti-chikungunya virus IIFT; Euroimmun, Germany). All patients underwent clinical examinations by experienced rheumatologists. We compared the frequency of chronic musculoskeletal manifestations and comorbidities in the two groups of patients: CHIKV-IgM-positive and CHIKV-IgM-negative. Data were analyzed using SPSS software and Fisher’s exact test. The significance level was set at p<0.05.

Between 12 and 36 months after CHIK acute infection, seven of 57 patients (12.3%) presented detectable CHIKV-IgM with a median time of 28.3 months (range, 15-35 months). Six of seven patients (86%) who presented with positive CHIKV-IgM reported persistent musculoskeletal symptoms. All these patients displayed joint pain, and two experienced persistent arthritis. The descriptive characteristics of the patients with persistent detectable CHIKV-IgM are shown in Table 1.

**TABLE 1:** Characteristics of patients with long-term detectable serum-specific anti-chikungunya IgM antibodies (CHIKV-IgM) at the Hospital das Clínicas, Universidade Federal de Pernambuco, Brazil.

Patients	Demographics^1^	Comorbidities^2^	Symptoms in acute phase	Persistent joint pain	Time since acute infection (months)
1	63y, M, White	MG, HTN	Fever/joint pain	Yes	15
2	50y, F, Nonwhite	HTN, DM	Fever/joint pain/rash	No	23
3	60y, F, Nonwhite	HTN, DM	Fever/joint pain	Yes	24
4	69y, F, Nonwhite	HTN	Joint pain/rash	Yes	32
5	51y, F,Nonwhite	-	Fever/joint pain/rash	Yes	32
6	44y, F, Nonwhite	HTN	Fever/joint pain/rash	Yes	34
7	39y, F, White	-	Fever/joint pain	Yes	35

^1^ Age (years); Sex (M=male, F=female), race; ^2^MG: membranous glomerulopathy; **HTN:** hypertension; **DM:** diabetes mellitus.

Patients positive for CHIKV-IgM were compared to those without detectable antibodies with respect to the presence of chronic musculoskeletal manifestations and underlying previous disease. The descriptive characteristics of the patients are presented in Table 2. Most of the 57 patients were female (86%), with a mean age of 52.2 years (SD, 11.4). The mean period since CHIK diagnosis was 31.8 months (SD, 5.7). Approximately 53% of the patients presented with underlying conditions, mainly hypertension and diabetes. In this sample, no differences were detected between the groups with positive or negative CHIKV-IgM in terms of the frequency of patients with persistent chronic musculoskeletal symptoms (86% × 82%, p = 0.76) or the presence of previous underlying chronic conditions (71% × 50%, p = 0.25) (Table 2). 

**TABLE 2:** Characteristics of 57 patients with persistent Chikungunya fever (CHIK) for more than 12 months, monitored at the Hospital das Clínicas, Universidade Federal de Pernambuco, Brazil.

Characteristics^1^	All patients	CHIKV-IgM positive	CHIKV-IgM negative
	(N = 57)	(N=7)	(N=50)
**Age, years** ^2^	52.2 (11.4)	53.7 (10.7)	52.0 (11.6)
**Sex**			
Female, N (%)	49 (86%)	6 (85.7%)	43 (86.0%)
Male, N (%)	8 (14%)	1 (14.3%)	7 (14.0%)
**Race**			
White, N (%)	17 (29.8%)	1 (14.3%)	16 (32.0%)
Nonwhite, N (%)	40 (70.2%)	6 (86.7%)	34 (68.0%)
**Time (in months) of CHIKV-IgM assessment after CHIK acute phase** ^2^	31.8 (5.7)	28.3 (7.7)	32.3 (5.3)
Comorbidities, N (%)	30 (52.6%)	5 (71.4%)	25 (50.0%)
Hypertension, N (%)	24 (42.1%)	5 (71.4%)	19 (38.0%)
Diabetes mellitus, N (%)	10 (17.5%)	2 (28.6%)	8 (16.0%)
Hypothyroidism, N (%)	5 (8.8%)	0 (0.0%)	5 (10.0%)
**Persistent chronic musculoskeletal symptoms, N (%)**	47 (82.4%)	6 (85.7%)	41 (82.0%)

^1^ p-value>0.05 was established for all characteristics; ^2^mean (SD). **CHIK:** chikungunya fever; **CHIKV-IgM:** serum-specific anti-chikungunya IgM antibodies.

## DISCUSSION

CHIK was first observed in Brazil in the Northern and Northeastern regions. Northeastern Brazil was severely affected by the epidemic, and it was necessary to establish a specific outpatient clinic for CHIK at the Hospital das Clínicas at the Universidade Federal de Pernambuco due to the high frequency of chronic manifestations. This led us to assess whether the long-term progression of CHIK in Brazilian patients was different from that in patients from other affected countries. Herein, we present the serological and clinical data of patients with long-term detectable CHIKV-IgM followed in a specific outpatient clinic for people with chronic symptoms after CHIK in Northeastern Brazil.

According to initial studies, after CHIKV infection, there is a progressive reduction in the production of CHIKV-IgM, which was detected in 76.2% of patients between two and six months, 69.8% between seven and 12 months, and 55.5% between 13 and 18 months after the acute phase^4^. Subsequent studies carried out in different regions of the world have demonstrated that CHIKV-IgM production may vary according to the time lapse between acute infection and the assessed population (Table 3). In Africa, CHIKV-IgM could be detected in up to 51% of patients approximately one year after CHIK, while Italy reported lower percentages of detectable antibodies, ranging from 13% to 17%, after a similar period^6^ ^-^ ^8^. In this study, we observed a frequency of CHIKV-IgM positivity comparable to that in Italian studies (12%), although with assessments performed at different periods. 

**TABLE 3:** Persistence of serum-specific IgM-chikungunya antibodies (CHIKV-IgM) in studies according to the time elapsed after chikungunya fever and the country of evaluation.

Author, year	Country	Time (months)^1^	CHIKV-IgM+ patients/total (%)
Grivard et al., 2007 [4]	Ilhas Reunion	13−18	25/45 (55.5)
Chopra et al., 2008 [10]	India	3−6	19/30 (63)
Oliver et al., 2009 [5]	France	13	2/18 (11.1)
Demanou et al., 2010 [6]	Cameroon	12	54/105 (51.4)
Moro et al., 2012 [7]	Italy	12−13	30/227 (13.2)
Pierro et al., 2015 [8]	Italy	12−13	23/133 (17.3)
Chelluboina et al., 2019 [9]	India	12	4/21 (19)

^1^time elapsed after chikungunya fever; **CHIKV-IgM:** serum-specific anti-chikungunya IgM antibodies.

It is plausible to accept that the difference observed in the present study may only be related to the time elapsed since the acute phase of CHIK as our assessment occurred after a mean interval of 28 months, which is longer than the intervals in previous studies. Furthermore, a commercially available ELISA kit was used in the present study; this may have reduced the sensitivity for detecting such antibodies^1^. We may also speculate that the long-term production of CHIKV-IgM could be related to the CHIKV genotype, host genetic factors, or epigenetic factors related to the environment, in addition to possible continuous antigenic stimulation.

Whether the persistence of CHIKV-IgM in the chronic phase of CHIK is related to a possible pathogenic role has not yet been fully established. Studies that only assessed individuals with persistent joint pain in the chronic phase of CHIK have demonstrated detectable CHIKV-IgM in around 63% after six months and in 11−19% after one year^5^ ^,^ ^9^ ^,^ ^10^. CHIKV-IgM was also detected in patients who had destructive arthritis up to three years after CHIKV infection, corroborating the hypothesis that it is persistently produced due to the maintenance of a tissue reservoir in the host^10^ ^,^ ^12^ ^,^ ^15^. Although our small sample size does not allow definitive conclusions, our results are similar to those of other studies that did not report a correlation between the presence of CHIKV-IgM and chronic joint manifestations of CHIK. For example, Moro and Borgherini detected CHIKV-IgM in 40% of patients up to 18.7 months after infection, with no association with persistent arthralgia^7^ ^,^ ^11^.

In addition, some studies have controversially reported that the severity of CHIK, as well as the chronicity of symptoms, may be related to the presence of comorbidities, such as hypertension and diabetes^13^ ^,^ ^14^. However, no studies have assessed whether the presence of these comorbidities affects the duration of IgM production. In our study, the frequency of underlying previous conditions did not differ between patients with positive and negative CHIKV-IgM, which may suggest that the presence of comorbidities is not related to the persistent production of these antibodies in the long-term.

In summary, in contrast to most studies that have analyzed patients up to 12−18 months post-CHIKV infection, this case series detected CHIKV-IgM for up to 35 months in 12.3% of Brazilian patients. The presence of persistent joint symptoms and underlying conditions did not seem to be associated with the long-term production of CHIKV-IgM in this sample, although further investigations are required to validate these observations. This study has indicated that a positive serological test for CHIK-IgM alone should not define the diagnosis of a recent disease.
